# Prevalence and incidence rates of 17 neuromuscular disorders: An updated review of the literature

**DOI:** 10.1177/22143602241313118

**Published:** 2025-03-04

**Authors:** Johanna CW Deenen, André LM Verbeek, Jan JGM Verschuuren, Baziel GM van Engelen, Nicol C Voermans

**Affiliations:** 1 Department of Neurology, Radboud university medical center, PO Box 9101, 6500 HB Nijmegen, the Netherlands; 2 Department for Health Evidence, Radboud university medical center, PO Box 9101, 6500 HB Nijmegen, the Netherlands; 3 Department of Neurology, Leiden University Medical Center, PO Box 9600, 2300 RC Leiden, the Netherlands

**Keywords:** neuromuscular disease, muscular diseases, epidemiology, incidence, prevalence

## Abstract

**Background:**

Epidemiological frequency measures serve as reference point for patients, clinicians, researchers, and policymakers. Previously, we published a comprehensive review of the literature with prevalence and incidence rates for thirty neuromuscular disorders frequently encountered in the neuromuscular clinic. No meta-analyses were available at the time.

**Objective:**

We included various new studies and meta-analyses that have been published since 2014, we aim to update our previous review.

**Methods:**

Pubmed was searched for ‘incidence’ and ‘prevalence’ in combination with seventeen acquired and inherited neuromuscular disorders to identify peer-reviewed literature from 1990 to 2023. If multiple prevalence and incidence rates were found, these were summarized by providing the mean, the number of the estimates on which the mean was based and the range of these estimates. Additionally, we searched for meta-analyses to compare the found mean prevalence rates based on the summary of individual studies with the pooled prevalence rates based on the meta-analyses.

**Results:**

The mean prevalence estimates for 17 disorders ranged from 0.3/100,000 population for Lambert-Eaton myasthenic syndrome, glycogenosis type V and nemaline myopathy to 20/100,000 for Charcot-Marie-Tooth disease type I. We found annual incidence rates for eight disorders, ranging from 0.3/100,000 population for progressive (spinal) muscular atrophy and facioscapulohumeral muscular atrophy to 1/100,000 for Charcot-Marie-Tooth disease type 1 and myotonic dystrophy type 1. Plotting the mean prevalence estimates from the current study against the pooled prevalence estimates from eight meta-analyses showed reasonable agreement.

**Conclusions:**

Epidemiological frequencies about neuromuscular diseases- and in particular data on incidence are scarce. The mean prevalence estimates based on recently published studies on individual cohorts correspond well with the findings from the sparingly performed meta-analyses.

## Introduction

The group of neuromuscular disorders (NMDs) comprises many different, individually rare diseases, but includes a large number of patients. As disease mechanisms are elucidated at a fast pace, clinical trials are being initiated for a growing number of disorders. Here, descriptive epidemiology serves as reference point for researchers as well as patients and clinicians. Epidemiological frequencies are not always readily available. This is especially the case for the rarer types of neuromuscular disorders.

In 1991, Emery published the first survey of the world literature with population frequencies of various neuromuscular disorders.^1,2^ We published in 2015 a literature review in order to update the epidemiological disease frequencies for several neuromuscular disorders covering the period from 1990 to June 2014.^3^

No systematic reviews or meta-analyses regarding the estimates of disease frequencies, including incidence and prevalence, were available at that time. The current study was performed to enable comparison with and consider completeness of a nationwide registry of newly diagnosed neuromuscular disorders in the Netherlands. It provided grouped epidemiological information and was also meant to serve as a reference point for patients, clinicians, researchers, and policymakers. We searched Pubmed using a simple search strategy and used a number of in- and exclusion criteria to serve as quality assessment. The choice for this search strategy was motivated by the sheer number of rare diseases we aimed to research: thirty neuromuscular disorders on multiple epidemiological aspects such as prevalence, incidence, age at onset and sex distribution.

**Figure 1.**
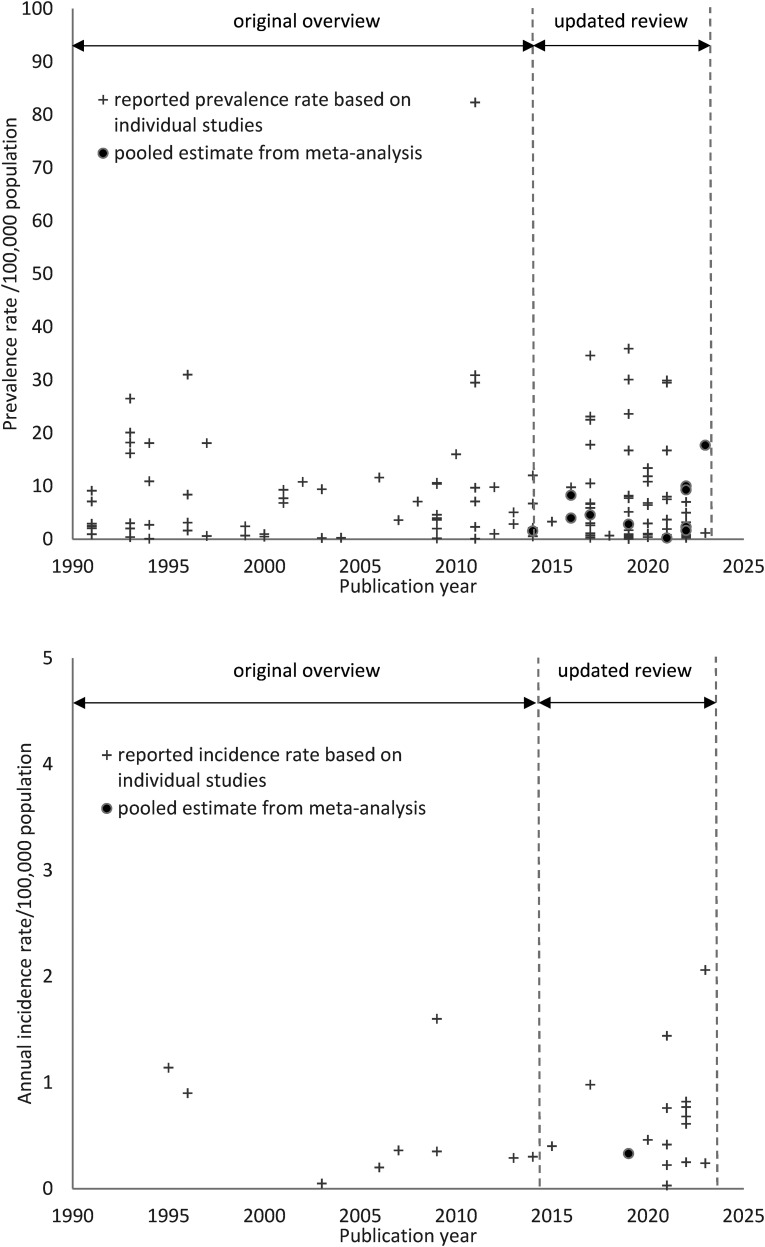
This figure shows all identified studies presenting one or more disease frequency estimates (incidence or prevalence rate) for the researched 17 neuromuscular disorders, each depicted as a plus. Identified pooled incidence or prevalence rate estimates from meta-analyses are shown as dots. Note the lack of meta-analyses up until 2015, the (rare) occurrence of meta-analyses since 2015, the scarcity of research-based incidence estimates and an even greater lack of pooled incidence rates based on meta-analyses.

Since July 2014, various new studies with disease frequencies of the neuromuscular disorder diagnoses of interest were published (see Figure 1). Additionally, systematic reviews and meta-analyses have emerged in the literature for the more common neuromuscular disorders, providing estimate ranges (in systemic reviews) and pooled disease frequency estimates (in meta-analyses). However, these meta-analyses focused on prevalence estimates, whereas pooled incidence estimates were still scarce.^4^

The interest in accurate information on epidemiological disease frequency has increased considerably, not only for patients and researchers, but also in clinical care and public health. The aim of this study is to update our previous literature overview on the incidence and prevalence of neuromuscular disorders with a summary of the existing knowledge from 1990 until 2024. This will be helpful to identify the gaps in the current knowledge of disease occurrence. Furthermore, it will also enable comparison of the recent findings to the current body of knowledge.

## Methods

### Researched neuromuscular disorders

This study, albeit an extension of our previous paper, focused solely on incidence and prevalence rates for a number of specific neuromuscular disorders, and contrary to the previous work it did not report age- and sex distributions.^3^ The researched disorders were based on previous research.^5^ We limited the number of studied disorders to those passing a set of five criteria enhancing validity. These five criteria arose from either capture-recapture specifics or characteristics of the used databases, and comprised the following items: 1) the disorder is also diagnosed in adulthood, 2) the disorder is chronic, 3) the diagnosis is sufficiently specific, 4) the Spierziekten Nederland patients association is findable by patients with the specific disorder, and 5) the diagnosis is predominantly made or confirmed in university medical centres. Applying these criteria deemed incidence rates to be estimated based on two nationwide datasets sufficiently accurate for the reporting.^5^ This resulted in a list of 17 neuromuscular disorders presented in Table 1.

**Table 1.** The 17 neuromuscular disorders included in this study arranged by anatomical location and in alphabetical order, accompanied by their common abbreviations.

*Origin* Disorders (and search term)	Common abbreviation
*Anterior horn cells*	
progressive (spinal) muscular atrophy	P(S)MA
*Peripheral nerve*	
Charcot-Marie-Tooth disease/ hereditary motor and sensory neuropathy	CMT / HMSN
Charcot-Marie-Tooth disease type 1 / hereditary motor and sensory neuropathy type 1*	CMT1 / HMSN1
Charcot-Marie-Tooth disease type 2/ hereditary motor and sensory neuropathy type 2*	CMT2 / H MSN2
chronic inflammatory demyelinating poly(radiculo)neuropathy	CIDP
multifocal motor neuropathy	MMN
*Neuromuscular junction*	
Lambert-Eaton myasthenic syndrome	LEMS
*Muscle*	
Becker muscular dystrophy	BMD
facioscapulohumeral muscular dystrophy	FSHD
glycogen storate disease type 2 / Pompe disease	GSD-II
glycogen storage disease type 5 / McArdle disease	GSD-V
inclusion body myositis	IBM
myotonic dystrophy	MD
myotonic dystrophy type 1 / Steinert disease*	MD1
myotonic dystrophy type 2 / Proximal myotonic myopathy*	MD2/PROMM
nemaline myopathy*	-
oculopharyngeal muscular dystrophy	OPMD

* not researched in previous review.^3^

### Search strategy

Pubmed was searched for articles from July 2014 to October 2023 for the neuromuscular disorders mentioned. For the newly added (subtypes of) disorders nemaline myopathy, myotonic dystrophy types 1 and 2, and Charcot-Marie-Tooth disease/hereditary motor and sensory polyneuropathy types 1 and 2, we searched the period from January 1990 to October 2023 (spanning the time frame of our first review and the current extension).

The literature search was similar to our Pubmed search in 2014, here we included the disorders mentioned in Table 1 instead of 30 disorders previously researched. We left out the MeSH-terms as we expected these would increase the number of found papers enormously without delivering additional relevant information. We combined all search terms with the keywords ‘incidence’ and ‘prevalence’ in title and abstract. Full search details are provided in the Supplementary materials Table 1. To enable comparison with systematic reviews and meta-analyses, we searched Pubmed for the words “systematic”, “review”, “meta” and “analys*” in the titles. These were combined with the search terms for the specific disorders (Supplementary Table 1).

### Inclusion criteria

Articles were considered eligible for data extraction if they met the following inclusion criteria: 1) published in English, 2) concerning the general population, not (a) specific ethnic group(s), 3) containing original data on incidence or prevalence, 4) methods of ascertainment and calculation of disease frequency mentioned, and 5) in case of multiple publications of the same or overlapping data: inclusion of the most recently reported data only.

### Rating of quality of evidence

Inclusion criterion 4 (mentioned methods of ascertainment and calculation of disease frequency) also served as the only quality rating we performed. If research outcomes are abundantly available, there is room for turning down findings based on quality assessment. We were in a situation where findings were still limited or even non-existent, and did not aim to include a meta-analysis where this information was needed. Furthermore, bias was to be expected in all articles due to the observational character of the studies and known heterogeneity of findings within multiple countries. Therefore, we did not apply a risk of bias assessment tool.

### Synthesis

After application of the inclusion criteria, the remaining articles were scrutinised for incidence and prevalence rates, and these were extracted. Prevalence rates were standardised to units of 100,000 population and incidence rates to units of 100,000 population per year. To facilitate comparison, estimates that used the number of live births as denominator (birth prevalence or live-birth incidence) were excluded.

Based on the findings from the identified studies we presented the range of the rates and the number of estimates, and calculated the mean of the available rates. Means were rounded to one significant digit, thus reporting on the general order of magnitude rather than seemingly exact numbers.

The data supporting the findings of this study are available within the article and/or its Supplementary materials.

## Results

The searched period from 1990 to 2023 yielded 69 eligible publications, including 26 new publications not included in our previous review (Table 2 and Table S1).^3^ The various incidence and prevalence rates for the 17 disorders are presented in Table 3 and Figure 1. The seven mean incidence estimates previously unavailable in the earlier review paper were on progressive (spinal) muscular atrophy, Charcot-Marie-Tooth disease types 1 and 2, multifocal motor neuropathy, Becker muscular dystrophy, facioscapulohumeral muscular dystrophy and myotonic dystrophy type 1. The incidence rate estimates varied from 0.3/100,000 population per year for progressive (spinal) muscular atrophy and facioscapulohumeral muscular atrophy to 1/100,000 for Charcot-Marie-Tooth disease type 1 and myotonic dystrophy type 1.

**Table 2.** The 17 neuromuscular disorders included in this study arranged by anatomical location and in alphabetical order, accompanied by the papers reporting on their incidence and prevalence.

*Origin* Disorders (and search term)	Referred publications
*Anterior horn cells*	
progressive (spinal) muscular atrophy	^11^
*Peripheral nerve*	
Charcot-Marie-Tooth disease/ hereditary motor and sensory neuropathy	^9,12–25^
Charcot-Marie-Tooth disease type 1 / hereditary motor and sensory neuropathy type 1*	^12,13,17–21,23–25^
Charcot-Marie-Tooth disease type 2/ hereditary motor and sensory neuropathy type 2*	^12,13,17–21,24,25^
chronic inflammatory demyelinating poly(radiculo)neuropathy	^20,26–37^
multifocal motor neuropathy	^20,33,36,38^
*Neuromuscular junction*	
Lambert-Eaton myasthenic syndrome	^9,20,39–41^
*Muscle*	
Becker muscular dystrophy	^14,20,25,42–52^
facioscapulohumeral muscular dystrophy	^14,20,25,43,44,49–57^
glycogen storate disease type 2 / Pompe disease	^50,51^
glycogen storage disease type 5 / McArdle disease	^20,50–52^
inclusion body myositis	^58–67^
myotonic dystrophy	^14,39,43,44,49,51,52,68–71^
myotonic dystrophy type 1 / Steinert disease*	^20,25,49–52,56,72–73^
myotonic dystrophy type 2 / Proximal myotonic myopathy*	^25,49,51,52,73^
nemaline myopathy*	^[20,25,49,51]^
oculopharyngeal muscular dystrophy	^20,25,49–51^

* Newly researched neuromuscular (sub-types of) disorders.

**Table 3.** Mean incidence and prevalence rates per 100,000 population of 17 researched neuromuscular disorders and the number of studies they are based on, arranged by anatomical location and in alphabetical order. We found prevalence rate estimates for all researched disorders, whereas incidence rate estimates were only encountered for eight of them.

Disorder	Incidence rate			Prevalence rate		
	mean/100,000	range/100,000	based on # studies	Mean /100,000	range/100,000	based on # studies
*Anterior horn cells*						
progressive (spinal) muscular atrophy	0.3	0.222 −0.415	1*	0.5	0.480–0.602	1*
*Peripheral nerve*						
Charcot-Marie-Tooth disease / hereditary motor and sensory neuropathy	1	0.98 –1.44	2	20	9.7–82.3	15
Charcot-Marie-Tooth disease type 1/ hereditary motor and sensory neuropathy type 1	-	-	-	10	4.19–30.9	10
Charcot-Marie-Tooth disease type 2/ hereditary motor and sensory neuropathy type 2	-	-	-	9	2,1–29,5	9
chronic inflammatory demyelinating polyneuropathy	0.7	0.24–1.6	8	4	0.04–8.1	12
multifocal motor neuropathy	0.6	-	1	0.8	0.53–1.33	4
*Neuromuscular junction*						
Lambert-Eaton myasthenic syndrome	0.04	0.03 –0.048	2	0.3	0.25–0.40	5
*Muscle*						
Becker muscular dystrophy	-	-	-	1	0. 04–3.645	14
facioscapulohumeral muscular dystrophy	0.3	-	1	5	0.79–12	14
glycogen storage disease type 2 (Pompe disease)	-	-	-	0.3	0.24–0.31	2
glycogenosis type 5	-	-	-	0.7	0.35–0.94	5
inclusion body myositis	0.4	0.09–0.76	5	3	0.0679–7.06	8
myotonic dystrophy	-	-	-	10	7.1–26.5	11
myotonic dystrophy type 1	1	0.2–2.061	3	10	0.47–35.9	10
myotonic dystrophy type 2/ proximal myotonic dystrophy	-	-	-	2	0.17–6.8	5
nemaline myopathy	-	-	-	0.3	0.14–0.4	4
oculopharyngeal muscular dystrophy	-	-	-	0.6	0.05–1.9	5

− no findings; * two findings in one publication.

We found prevalence rate estimates for all 17 disorders under investigation, ranging from 0.3/100,000 population for Lambert-Eaton myasthenic syndrome, glycogenosis type V and nemaline myopathy to 20/100,000 for Charcot-Marie-Tooth disease type 1. When these prevalence rates were summed, the 17 disorders comprised of a prevalence rate of 48/100,000, which is approximately 1 in 2100 of the population.

**Figure 2.**
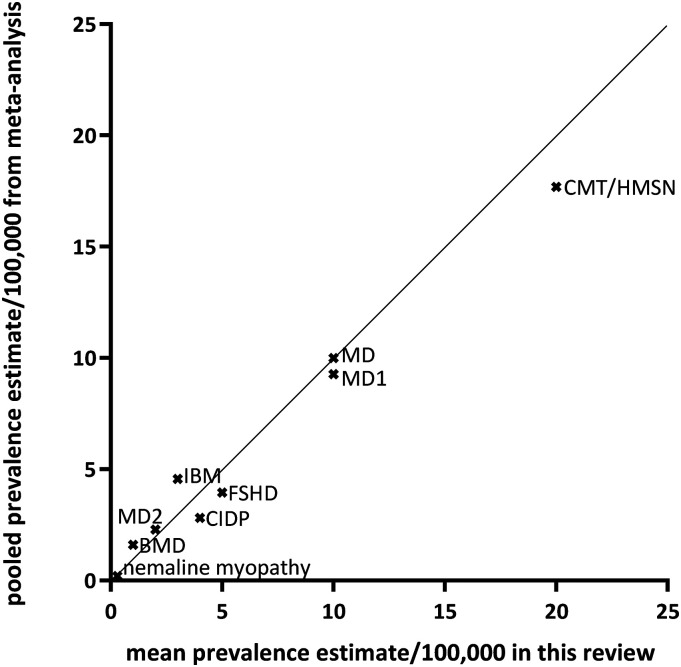
Comparison between meta-analysis-based pooled prevalence estimates per 100,000 population and the mean prevalence rates calculated from the individual studies found in this review for the neuromuscular disorders where a meta-analysis was available. BMD=Becker muscular dystrophy; CIDP=chronic inflammatory demyelinating polyneuropathy; CMT/HMSN=Charcot-Marie-Tooth disease / hereditary motor and sensory neuropathy; FSHD=facioscapulohumeral muscular dystrophy; IBM=inclusion body myositis; MD=myotonic dystrophy, types 1 and 2.

In addition, we identified eight publications providing one or more pooled prevalence estimates, see Table 4 and Figure 1, and one with a pooled incidence estimate. The lowest pooled prevalence estimate was calculated for nemaline myopathy: 0.20 per 100,000 population (95% CI, 0.10 −0.35). The highest estimate was for CMT/HMSN: 17.69 (95% CI, 12.32–24.33). The seven mean prevalence estimates from the current study were plotted against the pooled prevalence estimates from the meta-analyses, see Figure 2. A slightly oscillating pattern around the diagonal was noticed.

**Table 4.** Available pooled prevalence estimates per 100,000 population for 17 (groups of) neuromuscular disorders included in this study based on review and meta-analyses, arranged from highest to lowest mean prevalence estimate, with grouped subtypes. We found meta analyses or reviews for only half of the researched disorders. The available estimates were matched to the findings from this study to see how they relate.

	Current review		Meta-analyses		
Disorder	Mean estimate	Range	Pooled estimate	95% CI pooled estimate	Reference
					
MD	10	7.1–26.5	9.99*	5.62–15.53	^75^
			8.26	4.99–13.68	^76^
MD1	10	0.47–35.9	9.27	4.73–15.21	^75^
MD2	2	0.17–6.8	2.29	0.17–6.53	^75^
CMT/HMSN	20	9.7–82.3	17.69	12.32–24.33	^77^
CIDP	4	0.04–8.1	2.81	1.58–4.39	^4^**
IBM	3	0.0679–7.06	4.56	3.59–5.52	^78^
BMD	1 (/pop)	0.4–3.654 (/pop)	1.6 (/pop)	1.1–2.4 (/pop)	^79^
			1.53 (/male)	0.26–8.94 (/male)	^80^
FSHD	5	0.79–12	3.95	2.89–5.40	^76^
nemaline myopathy	0.3	0.14–0.4	0.20	0.10–0.35	^81^

* The most recent finding was used for comparison.

** One pooled incidence estimate was found for CIDP: 0.33/100,000/yr (95% CI, 0.21–0.53).

## Discussion

This review aimed to provide an update of valid grouped epidemiological information to both serve as a basis for comparison and as a reference point for patients, clinicians, researchers, and healthcare policymakers using studies of the last ten years. From the combined number of 68 articles published in 1990–2023, we calculated mean incidence rates for eight disorders ranging from 0.3 to 1.0 patients per 100,000 population per year. Eight meta-analyses have appeared in literature since our previous review.

In the last decade, the number of reports on incidence and prevalence estimates for various neuromuscular disorders has increased. This enables researchers to conduct meta-analyses. However, the number of meta-analyses was limited, especially on incidence estimates. We identified only one pooled incidence estimate: CIDP with a rate of 0.7 patients per 100,000 per year (95% CI, 0.21–4.39).^4^ Interestingly, it is more straightforward to obtain data on incidence than on prevalence, especially in the case of chronic disorders and even more so for disorders with limited or no treatment options. Newly diagnosed patients are easier to register than all patients who were ever diagnosed. Depending on (disease-)specific needs, people often become lost to follow-up, especially in chronic disorders and in the absence of treatment options. Furthermore, these patients might only have received a diagnosis based on clinical and / or histopathology features, without revision with currently available diagnostic methods (genetic testing, antibody screening). Moreover, incidence rates are more useful in the diagnostic process of rare diseases, because for prevalence, disease duration and therefore treatment effects need to be taken into account when using prevalence estimates.^6,7^ This is illustrated by the recent treatment developments in SMA, which will likely show an increase of disease duration in the near future due to the treatment success and an associated elevation of the prevalence.^8,9^

Although the screening of titles and abstracts and the assessment of full text was performed using a straightforward protocol, the current method has some disadvantages. Firstly, our search method was limited to Pubmed only and did not comprise other scientific sources such as Embase and CINAHL. This choice was driven by the need to research a large number of diseases. Therefore, we only used the database that encompasses the largest set of manuscripts and is most commonly used. Secondly, the screening of titles and abstract and of the full text was performed by a trained epidemiologist, and the methods were discussed extensively with a team of experienced epidemiologists and neurologists. Thirdly, references in identified manuscripts were not checked to upend other estimates, to prevent overlapping identified manuscripts. Finally, we neither took the number of cases nor the size of the population into account in the calculation of the summarizing means, as we were in search of the general order of magnitude of the estimates of interest.

For validity reasons, we compared the mean prevalence estimates from our current research with the sparsely available pooled prevalence outcomes of meta-analyses to assess the usefulness of these mean estimates in the absence of higher-level evidence. Despite the above-mentioned shortcomings, Table 4 and Figure 2 with the quantifications show considerable agreement between the findings of our simplified review exercise compared to the findings of the meta-analyses.

Patients, clinicians, researchers, and policymakers who are involved in neuromuscular disorders are interested in disease frequencies. However, due to the rarity of most neuromuscular disorders, accurate and representative epidemiological data are lacking. This review has shown that the number of publications on incidence and prevalence is rising, and even meta-analyses on disease frequency emerge amid the clinical literature. To monitor this trend and identify new and updated epidemiological information our PubMed searches appeared to be productive and our findings approached the available outcomes from meta-analyses remarkably well. Nonetheless, we prefer registry-based preferably nationwide data to fill the gaps in numerical disease data. Patient registries serve to overcome the research limitations inherent in the study of rare diseases, where patient numbers are typically small. Despite the value of real-world data collected through registries, adequate design and maintenance are integral to data quality.^10^

## Conclusion

The updated information on the incidence and prevalence of 17 neuromuscular disorders is specifically meant to enable comparisons in the absence of level-1 evidence from meta-analyses. High-quality studies based on nationwide (or wider) neuromuscular registries with clear standardized diagnostic criteria to estimate incidence and prevalence are expected to provide the required valid data.

## Supplemental Material

sj-docx-1-jnd-10.1177_22143602241313118 - Supplemental material for Prevalence and incidence rates of 17 neuromuscular disorders: An updated review of the literature Supplemental material, sj-docx-1-jnd-10.1177_22143602241313118 for Prevalence and incidence rates of 17 neuromuscular disorders: An updated review of the literature by Johanna CW Deenen, André LM Verbeek, Jan JGM Verschuuren, Baziel GM van Engelen and Nicol C Voermans in Journal of Neuromuscular Diseases
