# Supplementary material for: Prevalence and incidence rates of 17 neuromuscular disorders: An updated review of the literature
Source: J Neuromuscul Dis. 2025 Mar 4:22143602241313118. Online ahead of print. doi: 10.1177/22143602241313118 (PMC13142861; doi:10.1177/22143602241313118)
Supplement: Supplementary file 1 [file sj-docx-1-jnd-10.1177_22143602241313118.docx]

**SUPPLEMENTARY MATERIALS**

**Table 1. Search strategy for literature on descriptive epidemiological frequencies of the 17 neuromuscular disorders.**

For each neuromuscular disorder listed below, the provided search term was combined with:

1. **AND prevalence [tiab]**
2. **AND incidence [tiab]**
3. AND ((systematic[ti] AND review[ti]) OR (meta[ti] AND analys*[ti]))
4. AND ("1990/01/01"[Date - Publication] : "2023/10/01"[Date - Publication]) for newly researched disorders
5. AND ("2014/07/01"[Date - Publication] : "2023/10/01"[Date - Publication]) for previously researched disorders

MD

**myotonic dystrophy [tiab]**

MD1

**(**myotonic dystrophy [tiab] OR Steinert [tiab])

MD2

**(**myotonic dystrophy [tiab] OR PROMM [tiab])

CMT/HMSN

(hereditary motor sensory neuropathy [tiab] OR charcot marie tooth [tiab])

P(S)MA

(progressive spinal muscular atrophy [tiab]) OR (progressive muscular atrophy [tiab])

Pompe
(glycogenosis type 2 [tiab]) OR (glycogenosis type II [tiab]) OR (glycogen storage disease type 2 [tiab]) OR (glycogen storage disease type II [tiab]) OR (pompe [tiab])

MMN
multifocal motor neuropathy [tiab]

OPMD

oculopharyngeal muscular dystrophy [tiab]

BMD

becker muscular dystrophy [tiab]

Lambert-Eaton myasthenic syndrome

lambert eaton [tiab] OR eaton lambert [tiab]

chronic inflammatory demyelinating polyneuropathy

(chronic inflammatory demyelinating polyneuropathy [tiab] OR Chronic Inflammatory Demyelinating Polyradiculoneuropathy [tiab])

facioscapulohumeral muscular dystrophy

facioscapulohumeral muscular dystrophy [tiab]

nemaline myopathy

nemaline myopathy [tiab]

glycogenosis V

((glycogenosis type 5 [tiab]) OR (glycogenosis type V [tiab]) OR (glycogen storage disease type 5 [tiab]) OR (McArdle [tiab]))

IBM

Inclusion body myositis [tiab]

General

Neuromuscular [ti] AND disorder* [ti]

OR

Neuromuscular [ti] AND disease* [ti]

OR

Neurological [ti] AND disorder* [ti]

OR

Muscle [ti] AND disease* [ti]
