# Supplementary material for: Prevalence and incidence rates of 17 neuromuscular disorders: An updated review of the literature
Source: J Neuromuscul Dis. 2025 Mar 4;12(6):713–22. doi: 10.1177/22143602241313118 (PMC13142861; doi:10.1177/22143602241313118)
Supplement: sj-docx-1-jnd-10.1177_22143602241313118 - Supplemental material for Prevalence and incidence rates of 17 neuromuscular disorders: An updated review of the literature [file sj-docx-1-jnd-10.1177_22143602241313118.docx]

**SUPPLEMENTARY MATERIALS**

**Table 1. Search strategy for literature on descriptive epidemiological frequencies of the 17 neuromuscular disorders.**

| For each neuromuscular disorder listed below, the provided search term was combined with:   1. **AND prevalence [tiab]** 2. **AND incidence [tiab]** 3. AND ((systematic[ti] AND review[ti]) OR (meta[ti] AND analys*[ti])) 4. AND ("1990/01/01"[Date - Publication] : "2023/10/01"[Date - Publication]) for newly researched disorders 5. AND ("2014/07/01"[Date - Publication] : "2023/10/01"[Date - Publication]) for previously researched disorders   MD  **myotonic dystrophy [tiab]**  MD1  **(**myotonic dystrophy [tiab] OR Steinert [tiab])  MD2  **(**myotonic dystrophy [tiab] OR PROMM [tiab])  CMT/HMSN  (hereditary motor sensory neuropathy [tiab] OR charcot marie tooth [tiab])  P(S)MA  (progressive spinal muscular atrophy [tiab]) OR (progressive muscular atrophy [tiab])  Pompe (glycogenosis type 2 [tiab]) OR (glycogenosis type II [tiab]) OR (glycogen storage disease type 2 [tiab]) OR (glycogen storage disease type II [tiab]) OR (pompe [tiab])  MMN multifocal motor neuropathy [tiab]  OPMD  oculopharyngeal muscular dystrophy [tiab]  BMD  becker muscular dystrophy [tiab]  Lambert-Eaton myasthenic syndrome  lambert eaton [tiab] OR eaton lambert [tiab]  chronic inflammatory demyelinating polyneuropathy  (chronic inflammatory demyelinating polyneuropathy [tiab] OR Chronic Inflammatory Demyelinating Polyradiculoneuropathy [tiab])  facioscapulohumeral muscular dystrophy  facioscapulohumeral muscular dystrophy [tiab]  nemaline myopathy  nemaline myopathy [tiab]  glycogenosis V  ((glycogenosis type 5 [tiab]) OR (glycogenosis type V [tiab]) OR (glycogen storage disease type 5 [tiab]) OR (McArdle [tiab]))  IBM  Inclusion body myositis [tiab]  General  Neuromuscular [ti] AND disorder* [ti]  OR  Neuromuscular [ti] AND disease* [ti]  OR  Neurological [ti] AND disorder* [ti]  OR  Muscle [ti] AND disease* [ti] |
| --- |
